# OLDER INDONESIAN WOMEN’S EXPERIENCE TO MAINTAIN INDEPENDENCE IN FOOD-RELATED ACTIVITIES

**DOI:** 10.1093/geroni/igac059.1217

**Published:** 2022-12-20

**Authors:** Widya Ramadhani, Lynne Dearborn

**Affiliations:** University of Illinois Urbana-Champaign, Urbana, Illinois, United States; University of Illinois Urbana-Champaign, Champaign, Illinois, United States

## Abstract

The ability to conduct food-related activities such as cooking, eating, and cleaning are central to older adults’ health. Such ability is especially critical for older women who are more likely to live alone or become caregivers for their spouses and other family members. We investigated the experience of older Indonesian women, an understudied population in aging research, when engaging in food-related activities. Using the grounded theory approach, we examined the challenges and adaptive behavior of twelve community-dwelling older women in Indonesia (60+) when conducting food-related activities at home. We employed two data collection strategies: photo and video elicitation, followed by an interview. Through photo and video elicitation, participants took photos and videos of the space where they conducted food-related activities to provide physical environmental data. In the interview, participants explained their routine, activity challenges, and adaptation strategies in food-related activities. We discovered that participants’ view of their role in cooking food for the family is central to their sense of identity. When faced with age-related challenges, participants accepted assistance from others for activities less connected to food production, such as sweeping, mopping, or cleaning the kitchen. However, they are less likely to accept assistance for cooking activities. Instead, they adapted by modifying the physical environment, simplifying the tasks, and adjusting the method to remain engaged in cooking. Older Indonesian women’s cultural identity influenced their strategy to face age-related challenges and maintain independence. This finding highlights the importance of a culturally sensitive approach when planning support for older adults.

